# Impact of COVID-19 on the social relationships and mental health of older adults living alone: A two-year prospective cohort study

**DOI:** 10.1371/journal.pone.0270260

**Published:** 2022-07-06

**Authors:** So Im Ryu, Yeon-Hwan Park, Jinhyun Kim, Iksoo Huh, Sun Ju Chang, Soong-Nang Jang, Eun-Young Noh

**Affiliations:** 1 College of Nursing, The Research Institute of Nursing Science, Seoul National University, Seoul, Republic of Korea; 2 College of Nursing, Seoul National University, Seoul, Republic of Korea; 3 Red Cross College of Nursing, Chung-Ang University, Seoul, Republic of Korea; Gachon University Gil Medical Center, REPUBLIC OF KOREA

## Abstract

**Background:**

Owing to the COVID-19 outbreak, older adults living alone, who can only connect socially outside their homes, are at risk of social isolation and poor mental health. This study aimed to identify the changes, before and after COVID-19, by sex and age, in social relationships (social activity, social network, and social support) and mental health (depression and suicide ideation) among older adults living alone.

**Methods:**

This is a prospective cohort study of community-dwelling older adults who were at least 65 years old and living alone in South Korea. The study was conducted during 2018–2020 with 2,291 participants (795, 771, and 725 for the 1st to 3rd waves, respectively). The data were collected via face-to-face interviews. A generalized linear mixed modeling framework was used to test for changes over three years.

**Results:**

Social activity was reduced after the COVID-19, with an interaction effect of sex: older women (odds ratio [OR], 0.19; 95% confidence interval [CI], 0.15–0.23; *p* < .001) showed greater reduction than older men (OR, 0.50; 95% CI, 0.34–0.75; *p* < .001). Interaction with neighbors also reduced after the pandemic, but there was no significant evidence of interaction effects. Interaction with family members increased in both sexes during the pandemic, with the interaction effect of sex: older women (OR, 1.40; 95% CI, 1.11–1.76; *p* = .004) showed greater increase than men (OR, 1.55; 95% CI, 1.13–2.14; *p* = .007). Social support increased in both sexes during the pandemic, but there was no significant evidence of interaction effects. Depression and suicide ideation showed no significant differences before and after the pandemic.

**Conclusions:**

The findings provide health administrators and health providers with explorative insights into the impact of the COVID-19 on social relationships and mental health among older adults living alone and can guide further studies of interventions considering specific properties of social relationships.

## Introduction

A significant feature of population aging worldwide is the increasing proportion of older adults living alone, particularly evident in developed countries, including South Korea [1, 2]. “Living alone” is frequently considered an objective proxy of isolation; therefore, it is quite likely to increase the risk of social isolation due to a lack of opportunities for social contacts and networks [3, 4]. There has been robust evidence of the relationship between social isolation and health. Many studies reported that social isolation is associated with physical health, from immunological (i.e., increasing pro-inflammatory activity) to clinical effects (i.e., increasing the risk of coronary heart disease, stroke, and cancers) [4, 5]. Beyond physical health, social isolation can negatively impact cognitive function [6], mental health [7, 8], health-related behaviors [8, 9], and mortality [3]. Therefore, it is necessary to explore social relationships with sub-concepts among older adults living alone.

A social relationship generally comprises structural and functional aspects, which can be represented by social networks or activities and social support, although sub-concepts are controversial [10–12]. Many preceding studies have explored the concepts of social activity, social network, or social support with partial dimensions or a combined question [6, 13]. Similarly, other studies have adopted marital status, living alone, or their combined questions as indicators of social relationships. However, these are not adequate to understand the overall nature of social relationships [6, 14–17]. Therefore, for an in-depth understanding of social relationships among older adults living alone, it is necessary to explore the specific indicators of the concept separately and thoroughly.

Today, physical distancing has been encouraged to keep high-risk populations safe in countries affected by the rapid spread of COVID-19 [18]. Especially in South Korea, the first case occurred on January 20, 2020, which was followed by explosive outbreaks in February 2020. Consequently, the South Korean government activated a high-level health initiative to promote maintaining a distance of at least six feet, staying at home, and limiting face-to-face contact with others. However, older adults living alone, who are highly dependent on social welfare facilities to maintain their social relationships, face a greater risk of extreme social isolation during the shutdown period [19, 20]. To cope with social isolation effectively and efficiently during the current COVID-19 crisis or in similar public health crises in the future, it is necessary to ascertain what aspects of social relationships change by exploring situations before and after the crisis. However, to date, few studies have been conducted on this topic.

Traumatic events or social disruptions such as the COVID-19 crisis can affect mental health, particularly among those who are socially and economically vulnerable [21–24]. Contrary to expectations, some previous multinational studies have revealed that the number of suicides has remained unchanged or declined [25], and there are no significant changes for older adults [26]. However, these previous studies were limited in generalizing the result to older adults and older adults living alone, because there was no stratification according to age or the age group was not clear. Therefore, it is necessary to evaluate possible changes in depression and suicide ideation in older adults living alone, before and after the COVID-19 pandemic.

According to a narrative review about risk factors for suicide ideation and behavior among older adults, age-related illnesses and functional and physical declines may affect social isolation and depression, and these negative effects may lead to suicide ideations and behaviors [27]. Moreover, many empirical studies have suggested that there are sex-related differences in physical, functional, and mental health, as well as mortality including suicide [28]. Therefore, these factors need to be considered to establish rigorous evidence regarding social relationships and mental health.

Thus, this study aimed to identify the changes before (August 2018, 2019) and after the pandemic (August 2020) with a two-year follow-up, by sex and age, in social relationships and mental health among older adults living alone in South Korea.

## Methods

This study followed the Strengthening the Reporting of Observational Studies in Epidemiology or STROBE guidelines for cohort studies.

### Study design and setting

This was a prospective cohort study of three years (2018–2020) that tracked older adults (aged ≥ 65 years) living alone in S* City, South Korea. The first case of COVID-19 was reported on January 20, 2020, in South Korea. The incidence of COVID-19 peaked in mid-February 2020, and the second peak occurred on 13 August 2020 [29]. The Korean government tried to curb the crises by strengthening social distancing and reducing social activities including through a shutdown of social welfare facilities, day care centers, senior care facilities, and churches, on which older adults living alone rely [20, 30]. The 3rd wave of the cohort study was conducted from August 10, 2020 to August 21, 2020 in the middle of the second peak of the COVID-19 pandemic, which led to a strengthened social distancing policy in South Korea.

The study cohorts were derived from the project aimed to identify service needs and develop a community-based integrated service (CBIS) for older adults living alone [31]. The CBIS model is a tailored integrated care model for older adults living alone in South Korea. It is an age-friendly, integrated service system developed by the current research team. It comprises eight healthcare services, five social care services, and tailored case management; the intervention was implemented for six months (October 2019–April 2020; between the 2nd and 3rd wave cohorts, with a two-week break because of the COVID-19 outbreak) for cohort participants. The development process, pilot test results, and effectiveness of the model were presented in a previous report [32, 33].

### Participants

The cohorts were enrolled every August—in 2018 (1st wave), 2019 (2nd wave), and 2020 (3rd wave). Of the total number of older adults living in the 16 regions of S* City (3,753 older adults living alone as of 2018), 30% were recruited as participants in each period of the cohorts. When someone dropped out, newly enrolled participants were recruited by matching sex, age, and region to maintain the sample size. The inclusion criteria for the cohort study were as follows: 1) at least 65 years old, 2) living alone in S* City, and 3) able to communicate orally and provide written informed consent. Among the cohort participants, those who were included as the experimental group for applying the intervention of the CBIS model were excluded to avoid contamination bias.

**Fig 1:**
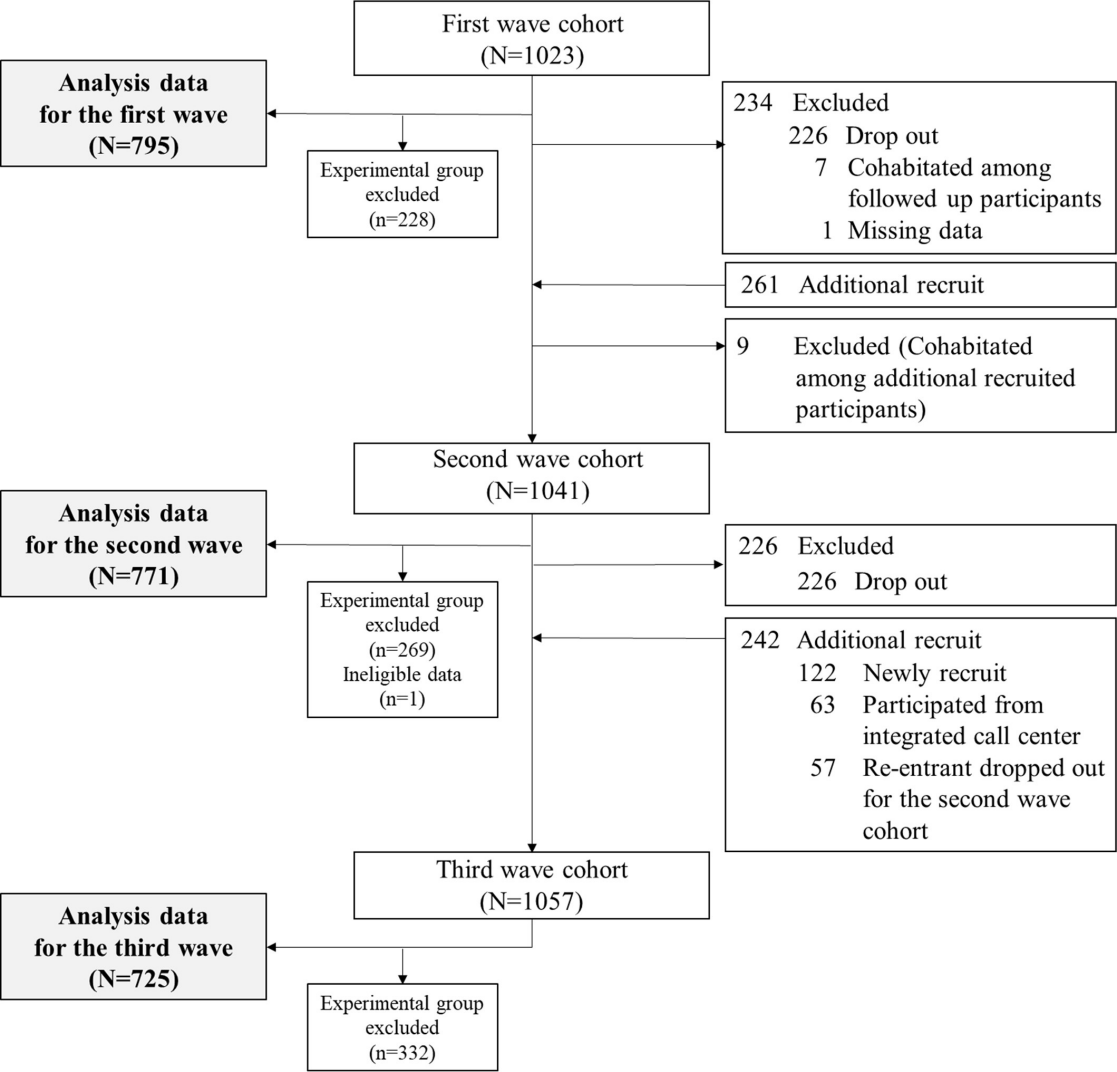
Flow chart for the process of cohort and analysis.

A total of 1,023 (1st wave), 1,041 (2nd wave), and 1,057 (3rd wave) participants were confirmed to be eligible for each cohort, and the processes of dropout and additional recruitment are described in Fig 1. The one-year follow-up rate was 77.1% from the 1st to 2nd waves and 78.2% from the 2nd to 3rd waves; the two-year follow-up rate from the 1st to 3rd waves was 63.8%. Most of the reasons for dropping out were personal issues, death, or being moved to another province. Finally, a total of 2,291 cohort participants (795 for the 1st wave, 771 for the 2nd wave, and 725 for the 3rd wave) were analyzed, excluding the experimental group.

### Measurement

#### Sociodemographic characteristics

Sociodemographic characteristics were measured as potential confounders. These characteristics included the following: age (65–74 years and ≥ 75 years); sex (male and female); presence of children living apart (yes and no); educational level (no formal education, elementary school, middle school, high school, and ≥ college); religion (yes and no); and personal income (< 980,000 Korean Won/month and ≥ 980,000 Korean Won/month), categorized based on the average income of older adults in South Korea in 2017 [34].

#### Social relationships

*Social activity*. Participation in social activity refers to the involvement in religious volunteer activities and leisure clubs, which is known as formal social participation [8, 35]. The frequency of participation in social activities was measured as a categorical variable (≥ 3–4 times/week, ≥ 1–2 times/week, ≥ 1–2 times/month, and none) by referring to the “Survey on the Current Status of Older Adults Living Alone,” which was used to select targets for “care services for older Koreans” [36]. As non-participation in social activities can be an indicator of social isolation, it was dichotomized into “none” and “≥ 1–2 times/month” based on a previous study [37].

*Social networks*. Social networks refer to the relationships between focal individuals surrounding a person that may or may not provide social support [10]. The frequency of interaction with neighbors (friends) and family members was measured as a categorical variable (≥ 1–2 times/week, ≥ 1–2 times/month, ≥ 1–2 times/quarter, ≥ 1–2 times/year, and none) by referring to the “Survey on the Current Status of Older Adults Living Alone” [36]. As older adults who contacted neighbors (friends) or family members less than once a month can be identified as socially isolated, it was dichotomized into less than monthly contact or more (< 1–2 times/month and ≥ 1–2 times/month) based on a previous study [37].

*Social support*. Social support is the functional aspect of social relationships, which refers to the perceived availability of social resources [4]. It was measured with the Korean version [38] of Enhancing Recovery in Coronary Health Disease Social Support Instrument [39]. These six self-reported items with binomial variables (yes and no) are scored from 0 to 6, with higher scores indicating higher social support. Cronbach’s alpha for the Korean version and the current study were .84 and .79, respectively.

#### Mental health

*Depression*. Depression was measured using the Korean version [40] of the Geriatric Depression Scale Short Form [41]. This is a 15-item, self-reported measurement that uses binomial variables (yes and no) ranging from 0 to 15, with higher scores indicating more depressive symptoms. In the Korean version [40] and the current study, the Cronbach’s alpha was .88.

*Suicide ideation*. Suicide ideation was measured using a 10-point visual analog scale. Participants in the study can respond to the question of “How often do you think about killing yourself?” with a score ranging from 0 (never) to 10 (very frequently) points.

### Data collection

The cohort data were collected through face-to-face interviews in August (August to October for the 1st wave) of each year from 2018 to 2020 at health or welfare centers in S* City, South Korea. Fifty assistants participated during the 1st wave (34 senior helpers in the community, called life managers, and 16 nursing college students), 69 for the 2nd wave (26 Red Cross volunteer groups, 35 nursing college and graduate students, and 8 health workers from S* Public Health Center for supporting the survey process), and 45 for the 3rd wave (37 nursing college and graduate students and 8 health workers from S* Public Health Center for supporting the survey process). All research assistants were trained for a minimum of two hours on research objectives, processes, questionnaire guidelines, and ethical considerations. The interview took approximately 40 minutes, and the participants who completed all the surveys received a small gift.

### Data analysis

Participants’ sociodemographic characteristics were shown as frequencies and percentages, and the chi-square test was used to test the homogeneity of proportions for three years. To explore the changes in outcome variables without adjustment, percentages for categorical variables and mean with standard error for continuous variables were used.

A generalized linear mixed modeling (GLMM) framework was used to test the changes in social relationships and mental health over three years. GLMM is an extension of the generalized linear model, which is effective for the longitudinal effects test with missing data to account for inter-subject heterogeneity and intra-subject correlation on repeated measures over the entire cohort. The impact of confounding covariates on the statistical model comes from both fixed and individual random effects. Thus, considering the data structure, including incomplete, longitudinal, and intra-subject dependency, GLMM was appropriate for this study to test the longitudinal effects.

Social relationships and mental health outcomes were defined as dependent variables, and sex, age, waves, and their interactions were considered fixed effects. Moreover, an unstructured covariance matrix was used for analysis. A GLMM model was fitted using normal distribution and the identity link function for continuous variables, and binomial distribution and the logit link function for categorical variables. The skewness values of social support, depression, and suicide ideation across the three waves are 0.30 to 0.35, 1.73 to 1.81, and -0.64 to -0.46, respectively. These can be considered as normally distributed. The analysis model for categorical outcomes (social activity, and interaction with neighbors, and interaction with family members) can be expressed as (1), and continuous outcomes (social support, depression, and suicide ideation) are expressed as (2):

$$\log\left(\frac{p_{ij}}{1-p_{ij}}\right)=\beta_{0}+\beta_{1}({Sex}_{i})+\beta_{2}({Age}_{i})+\beta_{3}({Time}_{j})+\beta_{4}({Sex}_{i}*{Time}_{j})+\beta_{5}({Age}_{i}*{Time}_{j})$$ (1)

$$y_{ij}=\beta_{0}+\beta_{1}({Sex}_{i})+\beta_{2}({Age}_{i})+\beta_{3}({Wave}_{j})+\beta_{4}({Sex}_{i}*{Wave}_{j})+\beta_{5}({Age}_{i}*{Wave}_{j})+\varepsilon_{ij}$$ (2)

i=1,…,N:participants,j=1,2,3:Wave

where *y* represents the dependent variable of individual *i* at wave *j*, and *β* denotes the estimated coefficient. Odds ratios (ORs) with 95% confidence intervals (CIs) were estimated to determine whether social relationships and metal health differ significantly across the waves. Sensitivity analysis was further performed to examine the results with fully observed data for the two-year follow-up and to determine the impact of attrition bias. The analysis was conducted in the same way as above. In this analysis, the GLIMMIX procedure in SAS (version 9.4; SAS Institute, Inc., Cary, NC, USA) and the R software (version 4.0.0; https://www.r-project.org/) were used to fit the model and figures. Statistical significance was set at *p* < .05.

### Ethical considerations

The study was reviewed and approved by the Institutional Review Board of the hospital of South Korea (approval no. H-1807-131-961). Researchers provided participants with information about the research and informed that there would be no disadvantages if they withdrew. Moreover, all participants provided signed informed consent.

## Results

### Sociodemographic characteristics

The sociodemographic characteristics of the participants and their homogeneity across the waves are presented in Table 1. The proportion of ≥ 75 years old increased from 60% in the 1st wave to 66% in the 3rd wave (*p* = .047), while there were no differences in the characteristics of participants throughout the waves. Most of the participants were female (77%), had more than one child living apart (91%), educated up to elementary school or less (≤ 6 years; 69%), and followed a religion (62–65%). Most of the participants’ income was lower than 980,000 Korean Won, which is the average income of older adults in South Korea.

**Table 1:** Sociodemographic characteristics for the three waves (N = 2,291).

Variables	*n* (%)			χ^2^ (p)
	1st Wave (n = 795)	2nd Wave (n = 771)	3rd Wave (n = 725)	
Age				6.097 (.047)
65–74 years old	318 (40.00)	292 (37.87)	246 (33.93)	
≥ 75 years old	477 (60.00)	479 (62.13)	479 (66.07)	
Sex				0.023 (.989)
Male	181 (22.77)	178 (23.09)	166 (22.90)	
Female	614 (77.23)	593 (76.91)	559 (77.10)	
Presence of children living apart				0.013 (.994)
Yes	726 (91.32)	704 (91.31)	661 (91.17)	
No	69 (8.68)	67 (8.69)	64 (8.83)	
Educational level (total years of schooling)				1.483 (.999)
No formal education	317 (39.87)	305 (39.56)	281 (38.76)	
Elementary school (6 years)	229 (28.81)	226 (29.31)	216 (29.79)	
Middle school (9 years)	115 (14.47)	108 (14.01)	107 (14.76)	
High school (12 years)	97 (12.20)	100 (12.97)	94 (12.97)	
≥ College (≥ 13 years)	37 (4.65)	32 (4.15)	27 (3.72)	
Religion				1.521 (.467)
Yes	503 (63.27)	502 (65.11)	450 (62.07)	
No	292 (36.73)	269 (34.89)	275 (37.93)	
Personal income^a^ (Korean Won/month)				0.489 (.793)
< 980,000	715 (90.05)	689 (89.48)	644 (88.95)	
≥ 980,000	79 (9.95)	81 (10.52)	80 (11.05)	

^a^ one missing data point

### Changes in social relationships and mental health

**Fig 2:**
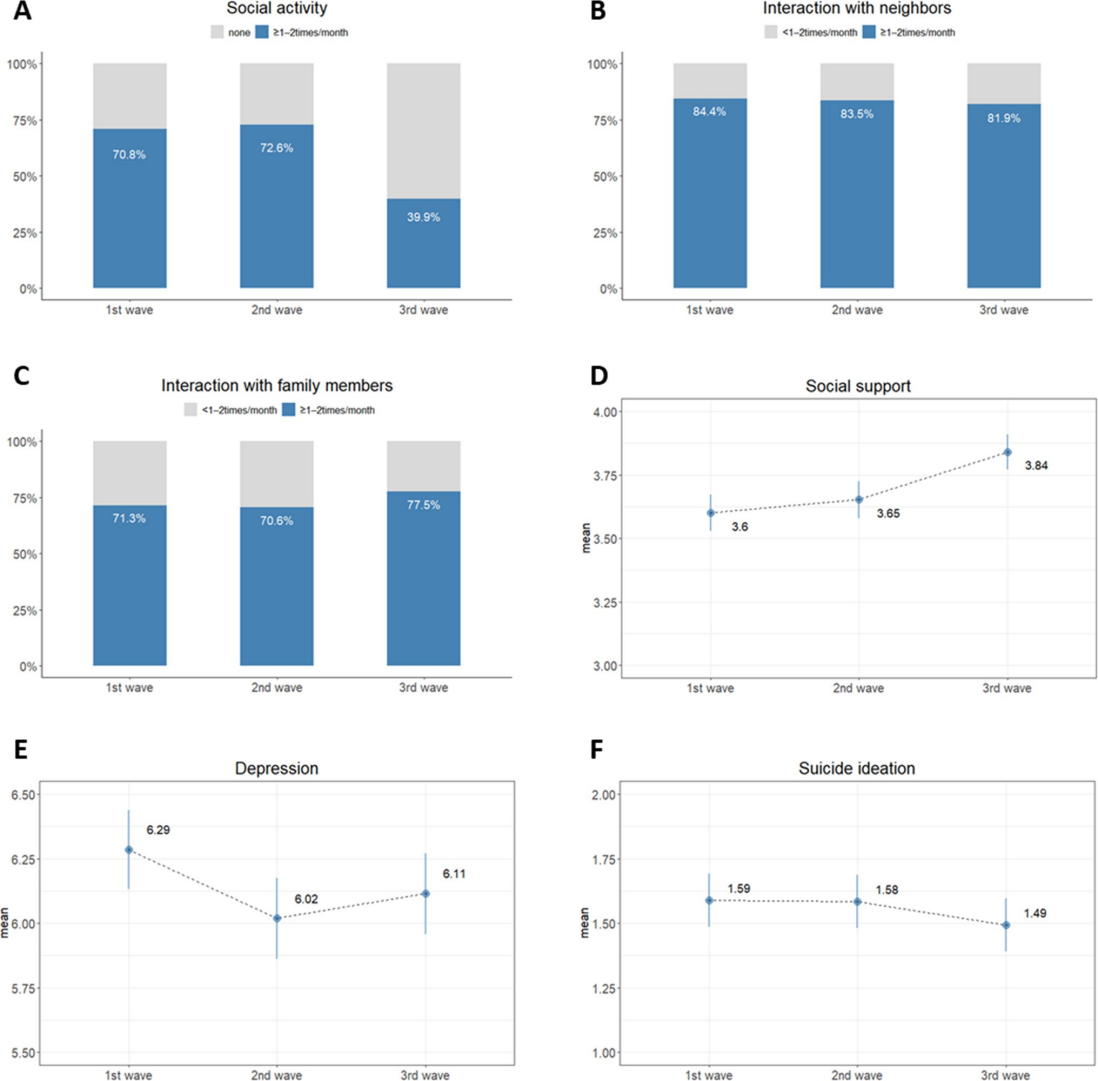
Social relationship and mental health change across waves.

Fig 2 shows the descriptive statistics for the outcome variables not adjusted for sex and age. There were reductions in participation in social activities (from 70.8% in the 1st wave to 39.9% in the 3rd wave) and interactions with neighbors (from 84.4% in the 1st wave to 81.9% in the 3rd wave). Conversely, there was an increase in the interactions with family members (from 71.3% in the 1st wave to 77.5% in the 3rd wave) and mean score of social support (from 3.60 ± 0.07 in the 1st wave, 3.65 ± 0.07 in the 2nd wave to 3.84 ± 0.07 in the 3rd wave).

Regarding mental health, the mean scores for depression and suicide ideation slightly decreased across the waves (*depression*: from 6.29 ± 0.15 in the 1st wave, 6.02 ± 0.16 in the 2nd wave to 6.11 ± 0.16 in the 3rd wave; *suicide ideation*: 1.59 ± 0.10 in the 1st wave, 1.58 ± 0.10 in the 2nd wave to 1.49 ± 0.10 in the 3rd wave).

### Changes in social relationship and mental health with interaction of sex and age

The results of estimating the changes in social relationships and mental health while considering the interaction effects of sex and age are presented in Tables 2 and 3 for categorical and continuous variables, respectively. Regarding categorical variables, social activity declined in the 3rd wave with the interaction effect of sex. The OR of older women’s social activities in the 3rd wave was 0.19 (95% CI, 0.15–0.23; *p* < .001) compared with the 1st wave and 0.18 (95% CI, 0.14–0.22; *p* < .001) compared with the 2nd wave. Further, the OR of older men’s social activities in the 3rd wave was 0.50 (95% CI, 0.34–0.75; *p* < .001) compared with the 1st wave and 0.43 (95% CI, 0.29–0.63; *p* < .001) compared with the 2nd wave. However, there was no significant evidence of changes between the 1st and 2nd waves for both sexes. The frequency of interaction with neighbors also decreased in the 3rd wave compared with the 1st wave adjusting for sex and age (OR, 0.77; 95% CI, 0.64–0.93; *p* = .006), but there was no significant evidence of interaction effects. The frequency of interaction with family members changed across the waves with the interaction effect of sex. The interaction with family members in older women decreased between the 1st and 2nd waves (OR, 0.80; 95% CI, 0.65–0.98; *p* = .033) but increased in the 3rd wave compared with the 1st (OR, 1.40; 95% CI, 1.11–1.76; *p* = .004) and 2nd waves (OR, 1.75; 95% CI, 1.41–2.17; *p* < .001), whereas it increased in the 2nd (OR, 1.43; 95% CI, 1.05–1.94; *p* = .025) and 3rd waves (OR, 1.55; 95% CI, 1.13–2.14; *p* = .007) in older men.

**Table 2:** Odds-ratio estimations from the generalized linear mixed modeling for categorical variables.

Variable	Wave						Age	Sex
	1st (ref) vs 2nd		1st (ref) vs 3rd		2nd (ref) vs 3rd		65–74 years (ref) vs ≥ 75 years	Male (ref) vs Female
	OR (95% CI)		OR (95% CI)		OR (95% CI)		OR (95% CI)	OR (95% CI)
	Male	Female	Male	Female	Male	Female		
Social activity	1.18 (0.88–1.59)	1.04 (0.85–1.26)	0.50*** (0.34–0.75)	0.19*** (0.15–0.23)	0.43*** (0.29–0.63)	0.18*** (0.14–0.22)	1.58*** (1.28–1.94)	-
Interaction with neighbors	0.95 (0.81–1.12)		0.77** (0.64–0.93)		0.81* (0.68–0.97)		1.35** (1.10–1.66)	3.04*** (2.44–3.79)
Interaction with family members	1.43* (1.05–1.94)	0.80* (0.65–0.98)	1.55** (1.13–2.14)	1.40** (1.11–1.76)	1.09 (0.80–1.48)	1.75*** (1.41–2.17)	1.71*** (1.40–2.10)	-

*p < .05

**p < .01

***p < .001

OR, odds ratio; 95% CI, 95% confidence interval

**Table 3:** Estimation of coefficients from generalized linear mixed modeling for continuous variables.

Variable	Fixed effects	β	SE	t or F (Num df)	*p-value*
Social support	(intercept)	2.867	0.120	23.91	< .001
	female	0.941	0.119	7.95	< .001
	≥ 75	0.021	0.098	0.22	.829
	wave			4.35 (2)	.013
	1(ref) vs 2	0.072	0.080	0.90	.367
	1(ref) vs 3	0.229	0.081	2.84	.005
	2(ref) vs 3	0.158	0.076	2.08	.038
Depression	(intercept)	6.748	0.271	24.89	< .001
	female	-0.398	0.282	-1.41	.158
	≥ 75	-0.209	0.219	-0.95	.341
	wave			1.97 (2)	.140
	1(ref) vs 2	-0.259	0.142	-1.83	.068
	1(ref) vs 3	-0.058	0.148	-0.39	.696
	2(ref) vs 3	0.201	0.135	1.49	.137
Suicide ideation	(intercept)	1.733	0.177	9.81	< .001
	female	-0.134	0.177	-0.76	.450
	≥ 75	-0.057	0.147	-0.39	.699
	wave			0.17 (2)	.842
	1(ref) vs 2	-0.022	0.112	-0.19	.846
	1(ref) vs 3	-0.068	0.119	-0.57	.567
	2(ref) vs 3	-0.046	0.112	-0.41	.680

SE, standard error; df, degree of freedom

**Fig 3:**
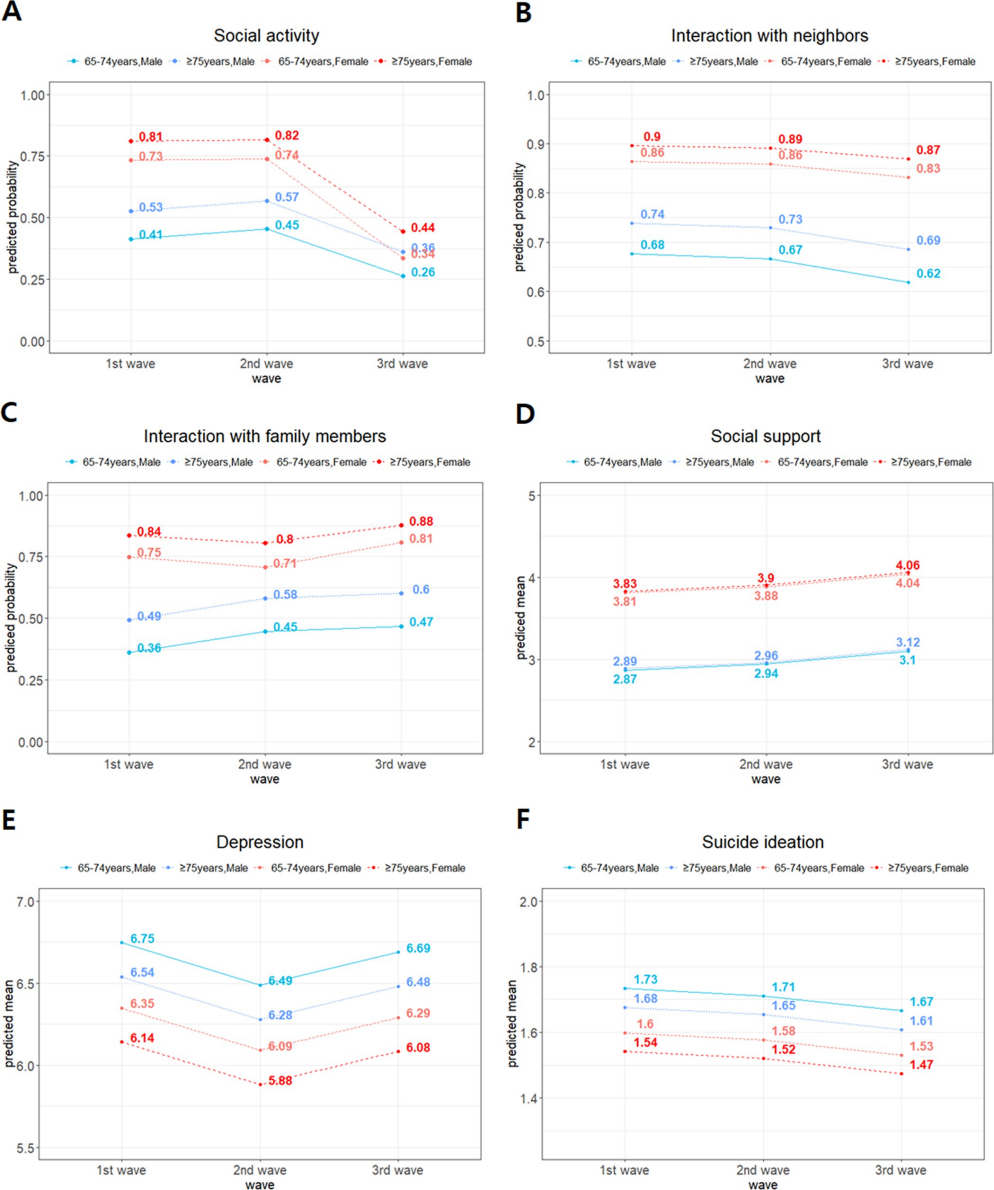
Changes in social relationships and mental health while considering the interaction effect. The (A) social activity and (C) interaction with family members showed significant interaction effects based on sex across the waves.

Regarding continuous variables, the social support levels increased in the 3rd wave compared with the 1st (β = 0.229, p = .005) and 2nd waves (β = 0.158, p = .038). This suggests that social support increased by 0.229 when changing from the 1st to 3rd wave and by 0.158 when changing from the 2nd to 3rd wave. However, there was no significant evidence of changes between the 1st and 2nd waves, and interaction effects. Moreover, there were no significant changes in the levels of depression and suicide ideation across the waves. Predicted probabilities and its trends across the waves in all variables are shown in Fig 3.

### Sensitivity analysis

When counting the number of participants excluding those who were newly included, 425 older adults participated in the two-year follow up (1st wave to 3rd wave). Considering the 795 older adults in the first primary survey (1st wave), the proportion of participants with two-year follow-up was 53.5%. Among the main results of the changes before and after the COVID-19 (2nd and 3rd wave), there were differences with respect to changes in social support and depression level. Contrary to the original results, there was no significant evidence of changes between the 2nd and 3rd waves in social support (β = 0.094, p = .311), but depression levels increased in the 3rd wave compared to the 2nd wave (β = 0.390, p = .013). All other main outcomes, including interaction effect and change before and after COVID-19 (2nd and 3rd wave), were similar to original results (S1 and S2 Tables).

## Discussion

This study investigated the changes, before and after the COVID-19 pandemic, in the social relationships and mental health of older adults living alone by considering the interaction effect of sex and age. The results showed that the change during a two-year follow-up period was significantly different among all the variables of social relationship—social activity, interaction with neighbors, interaction with family members, and social support. Among them, the interaction effect of sex was identified in social activities and interactions with family members. However, there was no significant evidence of changes in participants’ mental health, including depression and suicide ideation or age-related interaction effects before and after the COVID-19 outbreak.

The frequency of social activity was significantly reduced in both sexes during the pandemic, with greater reduction for older women than older men. This confirms the results of a qualitative study reporting decreased social activities, feeling of extreme isolation, and distress for being trapped at home alone among older adults living alone during the pandemic [19]. The finding of an interaction effect of sex across the waves corroborates a previous study reporting a greater decline in socializing and visiting public places among older women than older men [42]. Furthermore, Amagasa et al. [43] suggested that social activity at the community level is more important for older women than older men, because the former may experience greater sense of meaning and fulfillment in life than the latter. Therefore, in the face of the long-term pandemic, it is essential to develop interventions that encourage non-face-to-face technology-based social activity and verify its effectiveness.

Similarly, interaction with neighbors was significantly lower after COVID-19 (3rd wave) compared with the 1st and 2nd waves. This result can be understood by a previous study, wherein 28% of older adults living alone reported reduced contact with friends or neighbors, while 44% and 27% reported it to be the same as or more than before the COVID-19 outbreak, respectively [44]. The reason for this can be assumed to be the strengthened physical distancing policy. The survey on the 3rd wave was conducted when the pandemic reached its second peak. At that time, awareness of the pandemic was heightened, and the South Korean government raised the alert level while emphasizing social restraint [29]. Although direct comparison is difficult due to lack of information on the study period and the COVID-19 situation of the previous study, we can assume that the interaction with neighbors among older adults living alone decreased due to the sudden outbreak of COVID-19 and enhanced physical distancing.

We observed two pieces of evidence in terms of before and after the COVID-19 outbreak regarding the frequency of interaction with family members. First, before the outbreak, the frequency of interaction between older women living alone and their families decreased, while that between older men increased. To the best of our knowledge, the results cannot be compared to those of previous studies because of the rare longitudinal reports but may suggest potential implications. According to previous studies, older men living alone have shown poor health behavior (i.e., physical inactivity, smoking, and binge drinking) and a higher malnutrition rate than their female counterparts [45, 46]. Therefore, unlike in older women living alone, unhealthy habits and nutritional status of older men living alone may have raised family concerns over the well-being of older men living alone over time, which may have led to increased family contact. Second, after the outbreak, the frequency of interaction with family members increased for both sexes in the 3rd wave. This pattern is similar to the study of Fingerman et al. [44], which reports that most older adults living alone are in contact with families that is the same as before or more frequent. It can be speculated that the familial network function operates more frequently to share information or make safety checks during public health crises. However, older adults living alone with weak or absent familial networks may become more isolated during public health crises; therefore, they need to be supported by the management system more intensively.

Social support significantly increased during the COVID-19 pandemic, but there were no changes before the outbreak and no evidence of the interaction effects of sex or age. During the spread of COVID-19, while social support structures such as church and senior centers were shut down, S* City, where participants live, made efforts to support the emotional and daily life of older adults living alone (i.e., offering a kit for cultivating bean sprouts or pet plants, and food delivery service) directly through local governments or non-governmental organizations (https://www.siheung.go.kr/media/main.do). These direct and tailored efforts may make older adults living alone, amid the COVID-19 outbreak, feel supported.

Concerning mental health, depression and suicide ideation showed no significant changes before and after the COVID-19 outbreak, and there were no differences by sex and age. We need to discuss two aspects of the findings separately. First, the current findings regarding mental health associated with pandemic outbreaks contradict previous findings of a higher prevalence of depressive symptoms and suicide ideation after the COVID-19 outbreak [21, 47]. According to a previous study, social disconnectedness is one of the significant factors for perceived isolation, which, in turn, predicts depressive symptoms [48]. In other words, social connectedness can be a protective factor against depression and suicide during distressing situations among older adults [48, 49]. Considering the current results, social connectedness with family members and a high, perceived social support may relieve stress and play a role in buffering depressive symptoms and suicide ideation [50, 51]. Therefore, during a public health crisis, it is necessary to implement stay-at-home policies flexibly while maintaining social relationships and support. Additionally, a previous study revealed that older adults over 65 years old showed increased positive and decreased negative affect and felt less stress with their resilience during an unavoidable stressor such as the COVID-19 pandemic [52]. Therefore, it can be inferred that older adults’ resilience and ability to overcome stressful situations may have also influenced the mental health outcomes before and after the COVID-19 outbreak.

Furthermore, a two-year follow-up may be relatively short for identifying the resulting changes in mental health, which may appear after the actual pandemic [53]. Therefore, long-term follow-up research is needed to confirm the changes in depression and suicide ideation. Second, the current results showed no significant differences in depression and suicide ideation by sex and age, which is in contrast with previous studies [21]. The surveys in this study were conducted in different settings: the 1st and 2nd waves were conducted before the pandemic, and the 3rd wave was during the pandemic. Therefore, it is expected that the data collected in these different settings may have influenced the results of this analysis based on sex and age. Furthermore, the tendency to underreport depression and suicide ideation may have affected the result. Thus, caution is needed in interpreting the results.

The sensitivity analyses with full two-year follow up participants showed the robustness of the main findings. However, contrary to the original results, there were significantly more depressive symptoms, and evidence of increased social support after the COVID-19 outbreak was not found. The differences may be due to participant dropouts and new recruitments. It can be presumed that participants who dropped out were more likely to be socially isolated and depressed, whereas newly enrolled participants were more likely to be socially active and mentally healthy. In this case, missing data may not be random, and the incidence of adverse outcomes may be underestimated. Therefore, it is necessary to consider the possibility that the adverse outcomes of this study may have been underestimated when establishing strategies to improve social relationships and mental health in the context of a pandemic crisis.

### Limitations

Despite these findings, there are some limitations. First, although the GLMM framework was used to increase power and reduce bias in longitudinal studies with missing data, the sensitivity analyses suggested the potential attrition bias that was caused by dropouts and newly enrolled participants. Further, it is difficult to compare the social relationships and mental health of older adults living alone who had dropped out, because additional follow-up surveys were not conducted for such dropouts. Second, suicide ideation was measured using a simple and convenient visual analog scale, but it was not a structured measurement. Therefore, future studies need to explore the suicide ideation of older adults living alone with a structured and valid tool. Third, as the survey was conducted as a one-on-one interview in a face-to-face situation, there is a possibility that the participants gave false-negative responses on depression or suicide ideation due to worry about the social stigma. Thus, caution is required in interpreting the results because the participants’ tendency to underreport may affect the results. Fourth, despite a large sample size that is statistically adjusted for sex and age to establish external validity and unbiased results, there is potential for bias because this is a single city-based study. For robust generalization, further research is needed in multiple states, considering the proportion of older adults living alone.

## Conclusion

Amidst the pandemic (3rd wave), participation in social activities and interaction with neighbors reduced, but the interaction effect of sex was significant only for social activities. Interaction with family members and perceived social support increased in both sexes after the outbreak, but the interaction effect of sex was significant only for interaction with family members. However, there were no significant changes in depression and suicide ideation across the waves. This study can help develop an understanding of the changes in social relationships and mental health among older adults living alone before and after the COVID-19 crisis. Furthermore, it can be a cornerstone to guide further studies to design and plan successful interventions on social relationships among older adults living alone.

## Supporting information

S1 Table Odds-ratio estimations from the generalized linear mixed modeling for categorical variables with full two-year follow-up participants. (PDF)

S2 Table Estimation of coefficients from generalized linear mixed modeling for continuous variables with full two-year follow-up participants. (PDF)

S3 Table Description of sociodemographic characteristics for the three waves. (PDF)

S4 Table Description of social relationships and mental health for the three waves. (PDF)

## References

[1] Reher D, Requena M. Living alone in later life: a global perspective. Popul Dev Rev. 2018;44(3):427–454. doi: 10.1111/padr.12149

[2] Statistics Korea. Population projection for Korea [internet]. Seoul: KOSIS; 2019 Sep 18 [cited 2021 Nov 24]. Available from: http://kosis.kr/statHtml/statHtml.do?orgId=101&tblId=DT_1BZ0503&vw_cd=MT_ZTITLE&list_id=A42_10&seqNo=&lang_mode=ko&language=kor&obj_var_id=&itm_id=&conn_path=MT_ZTITLE.

[3] Holt-Lunstad J, Smith TB, Baker M, Harris T, Stephenson D. Loneliness and social isolation as risk factors for mortality: a meta-analytic review. Perspect Psychol Sci. 2015;10(2):227–237. doi: 10.1177/1745691614568352 25910392

[4] National Academies of Sciences, Engineering, and Medicine. Social isolation and loneliness in older adults: opportunities for the health care system. Washington, DC: The National Academies Press; 2020. 32510896

[5] Valtorta NK, Kanaan M, Gilbody S, Ronzi S, Hanratty B. Loneliness and social isolation as risk factors for coronary heart disease and stroke: systematic review and meta-analysis of longitudinal observational studies. Heart. 2016;102(13):1009–1016. doi: 10.1136/heartjnl-2015-308790 27091846 PMC4941172

[6] Evans IEM, Martyr A, Collins R, Brayne C, Clare L. Social isolation and cognitive function in later life: a systematic review and meta-analysis. J Alzheimers Dis. 2019;70(s1):S119–144. doi: 10.3233/JAD-180501 30372678 PMC6700717

[7] Smith KJ, Victor C. Typologies of loneliness, living alone and social isolation, and their associations with physical and mental health. Ageing Soc. 2019;39(8): 1709–1730. doi: 10.1017/S0144686X18000132

[8] Won S, Kim H. Social participation, health‐related behavior, and depression of older adults living alone in Korea. Asian Soc Work Policy Rev. 2020;14(1):61–71. doi: 10.1111/aswp.12193

[9] Cohen S, Gottlieb BH, Underwood LG. Social relationships and health. In: Cohen S, Underwood LG, Gottlieb BH, editors. Social support measurement and intervention: A guide for health and social scientist. New York: Oxford University Press; 2000 p. 3–28.

[10] Heaney CA, Israel BA. Social networks and social support. In: Glanz K, Rimer BK, Viswanath K, editors. Health behavior and health education: theory, research, and practice. San Francisco: Jossey-Bass; 2008. p. 189–207.

[11] House JS. Social support and social structure. Sociol Forum. 1987;2(1):135–146.

[12] Kuiper JS, Zuidersma M, Zuidema SU, Burgerhof JG, Stolk RP, Oude Voshaar RC, et al. Social relationships and cognitive decline: a systematic review and meta-analysis of longitudinal cohort studies. Int J Epidemiol. 2016;45(4):1169–1206. doi: 10.1093/ije/dyw089 27272181

[13] Santini ZI, Koyanagi A, Tyrovolas S, Mason C, Haro JM. The association between social relationships and depression: a systematic review. J Affect Disord. 2015;175:53–65. doi: 10.1016/j.jad.2014.12.049 25594512

[14] Holwerda TJ, Deeg DJH, Beekman ATF, van Tilburg TG, Stek ML, Jonker C, et al. Feelings of loneliness, but not social isolation, predict dementia onset: results from the Amsterdam Study of the Elderly (AMSTEL). J Neurol Neurosurg Psychiatry. 2014;85(2):135–142. doi: 10.1136/jnnp-2012-302755 23232034

[15] Steptoe A, Shankar A, Demakakos P, Wardle J. Social isolation, loneliness, and all-cause mortality in older men and women. Proc Natl Acad Sci USA. 2013;110(15):5797–5801. doi: 10.1073/pnas.1219686110 23530191 PMC3625264

[16] Holt-Lunstad J, Smith TB, Layton JB. Social relationships and mortality risk: a meta-analytic review. PLoS Med. 2010;7(7):e1000316. doi: 10.1371/journal.pmed.1000316 20668659 PMC2910600

[17] Sakurai R, Kawai H, Suzuki H, Kim H, Watanabe Y, Hirano H, et al. Poor social network, not living alone, is associated with incidence of adverse health outcomes in older adults. J Am Med Dir Assoc. 2019;20(11):1438–1443. doi: 10.1016/j.jamda.2019.02.021 31000349

[18] Lee H, Lee H, Song KH, Kim ES, Park JS, Jung J, et al. Impact of public health interventions on seasonal influenza activity during the COVID-19 outbreak in Korea. Clin Infect Dis. 2021;73(1):e132–140. doi: 10.1093/cid/ciaa672 32472687 PMC7314207

[19] Reher DS, Requena M, de Santis G, Esteve A, Bacci ML, Padyab M, et al. The COVID-19 pandemic in an aging world. 2020. doi: 10.31235/osf.io/bfvxt

[20] Jang SN, Kim CO. Care inequality among older adults during the COVID-19 pandemic. Ann Geriatr Med Res. 2020;24(4):229–231. doi: 10.4235/agmr.20.0096 33389970 PMC7781961

[21] Ettman CK, Abdalla SM, Cohen GH, Sampson L, Vivier PM, Galea S. Prevalence of depression symptoms in US adults before and during the COVID-19 pandemic. JAMA Netw Open. 2020;3(9):e2019686. doi: 10.1001/jamanetworkopen.2020.19686 32876685 PMC7489837

[22] Stahl ST, Beach SR, Musa D, Schulz R. Living alone and depression: the modifying role of the perceived neighborhood environment. Aging Ment Health. 2017;21(10):1065–1071. doi: 10.1080/13607863.2016.1191060 27267633 PMC5161727

[23] Xiu-Ying H, Qian C, Xiao-Dong P, Xue-Mei Z, Chang-Quan H. Living arrangements and risk for late life depression: a meta-analysis of published literature. Int J Psychiatry Med. 2012;43(1):19–34. doi: 10.2190/PM.43.1.b 22641928

[24] Almeida OP, Draper B, Snowdon J, Lautenschlager NT, Pirkis J, Byrne G, et al. Factors associated with suicidal thoughts in a large community study of older adults. Br J Psychiatry. 2012;201(6):466–472. doi: 10.1192/bjp.bp.112.110130 23209090

[25] Pirkis J, John A, Shin S, DelPozo-Banos M, Arya V, Analuisa-Aguilar P, et al. Suicide trends in the early months of the COVID-19 pandemic: an interrupted time-series analysis of preliminary data from 21 countries. Lancet Psychiatry. 2021;8(7):579–588. doi: 10.1016/S2215-0366(21)00091-2 33862016 PMC9188435

[26] Santomauro DF, Herrera AMM, Shadid J, Zheng P, Ashbaugh C, Pigott DM, et al. Global prevalence and burden of depressive and anxiety disorders in 204 countries and territories in 2020 due to the COVID-19 pandemic. Lancet. 2021;398(10312):1700–1712. doi: 10.1016/S0140-6736(21)02143-7 34634250 PMC8500697

[27] Conejero I, Olié E, Courtet P, Calati R. Suicide in older adults: current perspectives. Clin Interv Aging. 2018;13:691–699. doi: 10.2147/CIA.S130670 29719381 PMC5916258

[28] Oksuzyan A, Gumà J, Doblhammer G. Sex differences in health and survival. In: Gumà J, Doblhammer G, editors. A demographic perspective on gender, family and health in Europe. Cham (CH): Springer; 2018. p. 65–100.

[29] Seong H, Hyun HJ, Yun JG, Noh JY, Cheong HJ, Kim WJ, et al. Comparison of the second and third waves of the COVID-19 pandemic in South Korea: importance of early public health intervention. Int J Infect Dis. 2021;104:742–745. doi: 10.1016/j.ijid.2021.02.004 33556610 PMC7863747

[30] Ministry of Health and Welfare. IMS meeting for novel coronavirus presided over by the prime minister [internet]. Sejong (KR); 2020 Feb 13 [cited 2021 Nov 20]. Available from: https://www.mohw.go.kr/eng/nw/nw0101vw.jsp?PAR_MENU_ID=1007&MENU_ID=100701&page=1&CONT_SEQ=352865.

[31] Ko H, Park YH, Cho B, Lim KC, Chang SJ, Yi YM, et al. Gender differences in health status, quality of life, and community service needs of older adults living alone. Arch Gerontol Geriatr. 2019;83:239–245. doi: 10.1016/j.archger.2019.05.009 31102926

[32] Yi YM, Park YH, Cho B, Lim KC, Jang SN, Chang SJ, et al. Development of a Community-Based Integrated Service Model of health and social care for older adults living alone. Int J Environ Res Public Health. 2021;18(2):825. doi: 10.3390/ijerph18020825 33478027 PMC7835935

[33] Noh EY, Park YH, Cho B, Huh I, Lim KC, Ryu SI, et al. Effectiveness of a community-based integrated service model for older adults living alone: A nonrandomized prospective study. Geriatr Nurs. 2021;42:1488–1496. doi: 10.1016/j.gerinurse.2021.10.006 34706291

[34] Korea Institute for Health and Social Affairs. 2017 survey of living conditions and welfare needs of Korean older persons [internet]. Ministry of Health and Welfare of South Korea; 2018 [cited 2021 Nov 20]. Available from: http://www.mohw.go.kr/react/jb/sjb030301vw.jsp?PAR_MENU_ID=03&MENU_ID=032901&page=1&CONT_SEQ=344953.

[35] Donnelly EA, Hinterlong JE. Changes in social participation and volunteer activity among recently widowed older adults. Gerontologist. 2010;50(2):158–169. doi: 10.1093/geront/gnp103 19556394

[36] Ministry of Health Welfare [internet]. Care service for older adults. 2017 [cited 2021 Nov 20]. Available from: http://www.mohw.go.kr/react/jb/sjb030301vw.jsp?PAR_MENU_ID=03&MENU_ID=032901&CONT_SEQ=338646.

[37] Shankar A, McMunn A, Banks J, Steptoe A. Loneliness, social isolation, and behavioral and biological health indicators in older adults. Health Psychol. 2011;30(4):377–385. doi: 10.1037/a0022826 21534675

[38] Jeon GS, Jang SN, Park SJ. Social support, social network, and frailty in Korean elderly. J Korean Geriatr Soc. 2012;16(2):84–94. doi: 10.4235/jkgs.2012.16.2.84

[39] Mitchell PH, Powell L, Blumenthal J et al. A short social support measure for patients recovering from myocardial infarction: the ENRICHD social support inventory. J Cardiopulm Rehabil. 2003;23(6):398–403. doi: 10.1097/00008483-200311000-00001 14646785

[40] Kee BS. A preliminary study for the standardization of Geriatric Depression Scale Short Form-Korea version. J Korean Neuropsychiatr Assoc. 1996;35(2):298–306.

[41] Sheikh JI, Yesavage JA. Geriatric Depression Scale (GDS): recent evidence and development of a shorter version. Clin Gerontol. 1986;5(1–2):165–173. doi: 10.1300/J018v05n01_09

[42] Barber SJ, Kim H. COVID-19 worries and behavior changes in older and younger men and women. J Gerontol B Psychol Sci Soc Sci. 2021;76(2):e17–23. doi: 10.1093/geronb/gbaa068 32427341 PMC7313781

[43] Amagasa S, Fukushima N, Kikuchi H, Oka K, Takamiya T, Odagiri Y, et al. Types of social participation and psychological distress in Japanese older adults: a five-year cohort study. PLoS One. 2017;12(4):e0175392. doi: 10.1371/journal.pone.0175392 28388661 PMC5384679

[44] Fingerman KL, Ng YT, Zhang S, Britt K, Colera G, Birditt KS, et al. Living alone during COVID-19: social contact and emotional well-being among older adults. J Gerontol B Psychol Sci Soc Sci. 2021;76(3):e116–121. doi: 10.1093/geronb/gbaa200 33196815 PMC7717423

[45] Joo CL, Park JJ, Kim A, Park NL, Lim J, Park HS. Health behaviors and lifestyle patterns of elderly living alone in Korea. Korean J Fam Pract. 2019;9(3):247–253. doi: 10.21215/kjfp.2019.9.3.247

[46] WestergrenA, HagellP, Sjödahl HammarlundC. Malnutrition and risk of falling among elderly without home-help service—a cross sectional study. J Nutr Health Aging. 2014;18(10):905–911. doi: 10.1007/s12603-014-0469-5 25470807

[47] Killgore WDS, Cloonan SA, Taylor EC, Allbright MC, Dailey NS. Trends in suicidal ideation over the first three months of COVID-19 lockdowns. Psychiatry Res. 2020;293:113390. doi: 10.1016/j.psychres.2020.113390 32835926 PMC7430225

[48] Santini ZI, Jose PE, Cornwell EY, Koyanagi A, Nielsen L, Hinrichsen C, et al. Social disconnectedness, perceived isolation, and symptoms of depression and anxiety among older Americans (NSHAP): a longitudinal mediation analysis. Lancet Publ Health. 2020;5(1):e62–70. doi: 10.1016/S2468-2667(19)30230-0 31910981

[49] Wand APF, Zhong BL, Chiu HFK, Draper B, De Leo D. COVID-19: the implications for suicide in older adults. Int Psychogeriatr. 2020;32(10):1225–1230. doi: 10.1017/S1041610220000770 32349837 PMC7235297

[50] Lee HS, Kim C. Structural equation modeling to assess discrimination, stress, social support, and depression among the elderly women in South Korea. Asian Nurs Res (Korean Soc Nurs Sci). 2016;10(3):182–188. doi: 10.1016/j.anr.2016.06.003 27692246

[51] Purcell B, Heisel MJ, Speice J, Franus N, Conwell Y, Duberstein PR. Family connectedness moderates the association between living alone and suicide ideation in a clinical sample of adults 50 years and older. Am J Geriatr Psychiatry. 2012;20(8):717–723. doi: 10.1097/JGP.0b013e31822ccd79 22048322 PMC3276748

[52] Fields EC, Kensinger EA, Garcia SM, Ford JH, Cunningham TJ. With age comes well-being: older age associated with lower stress, negative affect, and depression throughout the COVID-19 pandemic. Aging Ment Health. 2021;1–9. doi: 10.1080/13607863.2021.2010183 34915781 PMC9200900

[53] Sher L. The impact of the COVID-19 pandemic on suicide rates. QJM. 2020;113(10):707–712. doi: 10.1093/qjmed/hcaa202 32539153 PMC7313777

