# Supplementary material for: Impact of COVID-19 on the social relationships and mental health of older adults living alone: A two-year prospective cohort study
Source: PLoS One. 2022 Jul 6;17(7):e0270260. doi: 10.1371/journal.pone.0270260 (PMC9258855; doi:10.1371/journal.pone.0270260)
Supplement: S1 Table — (PDF) [file pone.0270260.s001.pdf]

S1 Table. Odds-ratio estimations from the generalized linear mixed modeling for categorical variables with full three-year follow-up participants

(N = 425)

Variable	Wave						Age	Sex
	1st (ref) vs 2nd		1st (ref) vs 3rd		2nd (ref) vs 3rd		65–74 years (ref) vs ≥ 75 years	Male (ref) vs Female
	OR (95% CI)		OR (95% CI)		OR (95% CI)		OR (95% CI)	OR (95% CI)
	Male	Female	Male	Female	Male	Female		
Social activity	1.24 (0.84–1.84)	1.06 (0.83–1.36)	0.61 (0.37–1.01)	0.18*** (0.13–0.24)	0.49** (0.30–0.80)	0.17*** (0.13–0.22)	1.48** (1.10–2.00)	-
Interaction with neighbors	1.12 (0.92–1.37)		0.79* (0.62–0.99)		0.70** (0.56–0.87)		1.10 (0.81–1.48)	3.13*** (2.23–4.39)
Interaction with family members	1.47 (0.95–2.28)	0.63*** (0.49–0.81)	2.11*** (1.37–3.25)	1.32 (0.99–1.76)	1.43 (0.94–2.19)	2.10*** (1.60–2.76)	1.45* (1.08–1.94)	-

$^{*}p < .05$
$^{**}p < .01$
$^{***}p < .001$
OR, odds ratio; 95% CI, 95% confidence interval
