# Supplementary material for: Impact of COVID-19 on the social relationships and mental health of older adults living alone: A two-year prospective cohort study
Source: PLoS One. 2022 Jul 6;17(7):e0270260. doi: 10.1371/journal.pone.0270260 (PMC9258855; doi:10.1371/journal.pone.0270260)
Supplement: S2 Table — (PDF) [file pone.0270260.s002.pdf]

S2 Table. Estimation of coefficients from generalized linear mixed modeling for continuous variables with full three-year follow-up participants (N = 425)

Variable	Fixed effects	β	SE	t or F (Num df)	*p-value*
Social support	(intercept)	2.572	0.178	14.43	< .001
	female	1.149	0.183	6.27	< .001
	≥ 75	-0.030	0.139	-0.22	.828
	wave			5.45 (2)	.005
	1(ref) vs 2	0.237	0.098	2.41	.016
	1(ref) vs 3	0.331	0.102	3.23	.001
	2(ref) vs 3	0.094	0.093	1.01	.311
Depression	(intercept)	7.213	0.426	16.91	< .001
	female	-1.029	0.454	-2.27	.024
	≥ 75	-0.209	0.302	-0.69	.488
	wave			5.21 (2)	.006
	1(ref) vs 2	-0.505	0.175	-2.88	.004
	1(ref) vs 3	-0.115	0.182	-0.63	.529
	2(ref) vs 3	0.390	0.156	2.50	.013
Suicide ideation	(intercept)	1.810	0.260	6.96	< .001
	female	-0.408	0.268	-1.52	.128
	≥ 75	0.171	0.200	0.85	.395
	wave			0.80 (2)	.449
	1(ref) vs 2	-0.176	0.141	-1.25	.212
	1(ref) vs 3	-0.130	0.145	-0.90	.370
	2(ref) vs 3	0.046	0.132	0.35	.727

SE, standard error; df, degree of freedom
