# Supplementary material for: Impact of COVID-19 on the social relationships and mental health of older adults living alone: A two-year prospective cohort study
Source: PLoS One. 2022 Jul 6;17(7):e0270260. doi: 10.1371/journal.pone.0270260 (PMC9258855; doi:10.1371/journal.pone.0270260)
Supplement: S3 Table — (PDF) [file pone.0270260.s003.pdf]

S3 Table. Description of sociodemographic characteristics for the three waves

Variables	1st Wave (n = 795)			2nd Wave (n = 771)			3rd Wave (n = 725)		
	n (%)	Mean ± SD (Range)	SE	n (%)	Mean ± SD (Range)	SE	n (%)	Mean ± SD (Range)	SE
Age		76.00 ± 5.78 (65–99)	0.21		76.59 ± 5.49 (66–91)	0.20		77.06 ± 5.42 (66–95)	0.20
65–74 years old	318 (40.00)			292 (37.87)			246 (33.93)		
≥ 75 years old	477 (60.00)			479 (62.13)			479 (66.07)		
Sex									
Male	181 (22.77)			178 (23.09)			166 (22.90)		
Female	614 (77.23)			593 (76.91)			559 (77.10)		
Presence of children living apart									
Yes	726 (91.32)			704 (91.31)			661 (91.17)		
No	69 (8.68)			67 (8.69)			64 (8.83)		
Educational level (total years of schooling)									
No formal education	317 (39.87)			305 (39.56)			281 (38.76)		
Elementary school (6 years)	229 (28.81)			226 (29.31)			216 (29.79)		
Middle school (9 years)	115 (14.47)			108 (14.01)			107 (14.76)		
High school (12 years)	97 (12.20)			100 (12.97)			94 (12.97)		
≥ College (≥ 13 years)	37 (4.65)			32 (4.15)			27 (3.72)		
Religion									
Yes	503 (63.27)			502 (65.11)			450 (62.07)		
No	292 (36.73)			269 (34.89)			275 (37.93)		
Personal income[a] (Korean Won/month)		560,348 ± 421,762 (0–6,000,000)	14,967		604,674 ± 417,725 (0–6,000,000)	15,053		631,187 ± 360,839 (0–5,000,000)	13,410
< 980,000	715 (90.05)			689 (89.48)			644 (88.95)		
≥ 980,000	79 (9.95)			81 (10.52)			80 (11.05)		

[a] one missing data point

SD, standard deviation; SE, standard error
