# Supplementary material for: Impact of COVID-19 on the social relationships and mental health of older adults living alone: A two-year prospective cohort study
Source: PLoS One. 2022 Jul 6;17(7):e0270260. doi: 10.1371/journal.pone.0270260 (PMC9258855; doi:10.1371/journal.pone.0270260)
Supplement: S4 Table — (PDF) [file pone.0270260.s004.pdf]

S4 Table. Description of social relationships and mental health for the three waves

Variables	1st Wave (n = 795)			2nd Wave (n = 771)			3rd Wave (n = 725)		
	n (%)	Mean ± SD (Range)	SE	n (%)	Mean ± SD (Range)	SE	n (%)	Mean ± SD (Range)	SE
Social activity									
≥ 1–2 times/month	563 (70.82)			560 (72.63)			289 (39.86)		
none	232 (29.18)			211 (27.37)			436 (60.14)		
Interaction with neighbors									
< 1–2 times/month	124 (15.60)			127 (16.47)			131(18.07)		
≥ 1–2 times/month	671 (84.40)			644 (83.53)			594 (81.93)		
Interaction with family members									
< 1–2 times/month	228 (28.68)			226 (29.41)			163 (22.48)		
≥ 1–2 times/month	567 (71.32)			545 (70.59)			562 (77.52)		
Social support		3.60 ± 2.03 (0–6)	0.07		3.65 ± 2.01 (0–6)	0.07		3.84 ± 1.86 (0–6)	0.07
Depression		6.29 ± 4.33 (0–15)	0.15		6.02 ± 4.36 (0–15)	0.16		6.11 ± 4.22 (0–15)	0.16
Suicide ideation		1.59 ± 2.89 (0–10)	0.10		1.58 ± 2.85 (0–10)	0.10		1.49 ± 2.78 (0–10)	0.10

SD, standard deviation; SE, standard error
